# Ectopic breast tissue (polymastia)

**DOI:** 10.11604/pamj.2025.52.166.49034

**Published:** 2025-12-17

**Authors:** Pranjali Saranjami Jode, Deeplata Mendhe

**Affiliations:** 1 Community Health Nursing Department, Smt. Radhikabai Meghe Memorial College of Nursing, Sawangi (Meghe), Wardha, Maharashtra, India

**Keywords:** Ectopic breast tissue, polymastia, accessory breast, supernumerary breast

## Image in medicine

A 16-year-old girl was brought to the outpatient clinic by her parents due to a progressively enlarging, painless swelling on her upper back, which had been present since early childhood but became more noticeable during puberty. The swelling was soft, mobile, and had normal overlying skin with a distinct areola and nipple-like structure. There were no signs of inflammation, discharge, or tenderness. On examination, the mass measured approximately 6 cm in diameter and was located on the left paravertebral region. The presence of a nipple-areola complex over the swelling suggested a diagnosis of ectopic breast tissue (polymastia). There was no similar swelling in the normal pectoral area, and no associated lymphadenopathy was found. The patient had otherwise normal development and no significant medical or surgical history. Ectopic breast tissue occurs due to the failure of regression of the embryonic mammary ridge (milk line), which extends from the axilla to the groin. This condition is usually benign and can be found anywhere along the milk line, though occurrences on the back are extremely rare. It may undergo the same physiological changes as normal breast tissue, including enlargement during puberty, menstruation, pregnancy, and lactation. While typically asymptomatic, ectopic breast tissue carries a small risk of developing benign or malignant lesions. Management depends on the patient's symptoms and cosmetic concerns. In this case, the patient and her family opted for surgical excision for cosmetic reasons, and histopathology confirmed the presence of benign breast tissue.

**Figure 1:**
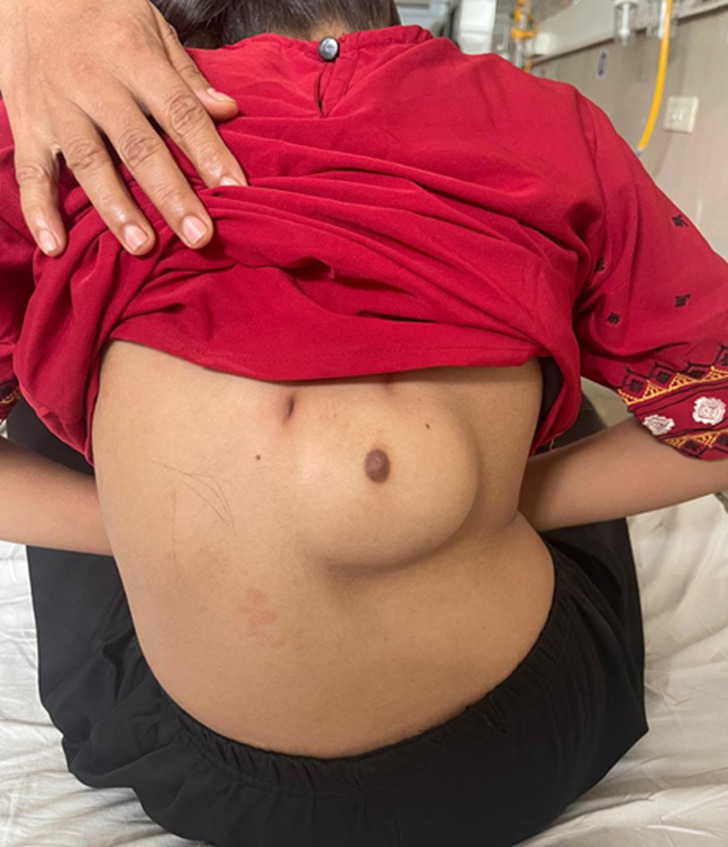
ectopic breast tissue (polymastia) located on the back with a visible nipple-areola complex

